# Development and Psychometric Evaluation of a Socioeconomic Status Questionnaire for Urban Households (SESIran): The Preliminary Version

**DOI:** 10.15171/hpp.2015.030

**Published:** 2016-01-30

**Authors:** Omid Abobakri, Homayoun Sadeghi-Bazargani, Mohammad Asghari-Jafarabadi, Mohammad Bagher Alizadeh Aghdam, Ali Imani, Jafarsadegh Tabrizi, Shaker Salarilak, Mostafa Farahbakhsh

**Affiliations:** ^1^Department of Biostatistics & Epidemiology, Tabriz University of Medical Sciences, Tabriz, Iran; ^2^Road Traffic Injury Research Center, Statistics & Epidemiology Department, Tabriz University of Medical Sciences, Tabriz, Iran; ^3^Medical Education Research Center, Tabriz University of Medical Sciences, Tabriz, Iran; ^4^Department of Social Science, University of Tabriz, Tabriz, Iran; ^5^Iranian Center of Health Management (IceHM), Department of Health Services Management, Tabriz University of Medical Sciences, Tabriz, Iran; ^6^Department of Health Services Management, Health Services Management Research Center, Tabriz University of Medical Sciences, Tabriz, Iran; ^7^Department of Public Health, Islamic Azad University of Tabriz Branch, Tabriz, Iran; ^8^Tabriz University of Medical Sciences, Tabriz, Iran

**Keywords:** Socioeconomic Status, SESIran, Health, Household, Validity, Reliability

## Abstract

**
Background:** The aim of present study was to develop and validate an appropriate socioeconomic status (SES) assessment questionnaire to be used through health studies in Iranian urban households.

**
Methods:** The study was conducted through a mixed method study design in Tabriz, northwest of Iran in 2014. It was conducted in several stages including: development of initial version, qualitative study, feasibility evaluation, and assessment of the validity as well as the reliability. The internal consistency, test-retest reliability, content validity, concurrent validity and construct validity were assessed.

**
Results:** With respect to the assessment of construct validity, 5 domains (factors) were extracted includ­ing: main factor (α=0.84), self-evaluation of expenditure capacity (α=0.96), wealth (α=0.70), home and furniture (α=0.66) and costs related to health (α=0.55). Intraclass correlation coefficient was above 0.6 for all factors except for wealth domain.

**
Conclusion:** The questionnaire developed appeared to be a valid and reliable SES assessment tool. It may be of value to be used not only as a complementary questionnaire in most health surveys or clini­cal studies, but also as a main questionnaire in health equity and health economics research.

## Introduction

According to WHO definition, “health is a state of complete physical, mental and social well-being and not merely the absence of disease or infirmity”.^1^ Over time, researchers have changed their view of health and turned their attention towards the ecological approach. Accordingly, human is considered as a subclass of social, economic, political, and environmental system and his or her health is affected by these factors.^2^ Socioeconomic Status (SES) is defined as someone’s or group's position within a hierarchical social structure and is combined of variables, including job, education, income, wealth, and place of residence.^3^ It is one of the variables considered in social and health studies.

Due to the influence of SES from the structure of society, culture, social relations and policies of society, its exact measurement is difficult to accomplish. Moreover, to the extent that communities distance themselves from extensive political, economic and social fluctuations, and to the extent that the rules of law are institutionalized, the measurement and ranking of SES becomes easier.^4^ Therefore, it is difficult to measure the variable in under developed countries and adequate tools are not often available in these countries. Assessing SES is easier to be done in a reliable way in developed countries than in less developed countries. Different studies have used different indicators to measure the socio-economic status. For example, education and occupation are used more frequently than level of income and assets. Nevertheless, it is important to note that level of income and assets have important and potential roles in determination of socioeconomic status.^5^

Much effort has been made in many studies conducted in Iran to measure SES mostly through single arbitrary questions on education or income, however, developing a questionnaire that enables measuring SES reliably in developing countries; especially in Iran is not well addressed. An instrument of socio-economic status assessment is developed and updated annually in India, which had three domains: employment, education and household.^6^ It classified the socioeconomic status in three levels (medium, high and low). Due to the continued economic inflation and decreasing value of the currency, researchers have updated only the domain of household’s income. Other questionnaires have also been developed. For instance, the questionnaire developed in 1983, which had benefits for researchers and was assessed by Gilany et al.^7^ They found that new scale was composed of 7 domains namely education, culture, occupation, family, family possessions, economic, home sanitation and health care.

Iran is a middle-income country that has made good progress in the field of health research. However, Iranian researchers have not evaluated the socio-economic status reliably in some of their studies. It makes difficult to interpret and draw highly valid conclusions in cases where SES is considered as a potential confounder or major variable of interest. Despite this, there exist a few SES assessments questionnaires in Eastern Mediterranean countries including Iran. The only questionnaire retrieved capable of assessing the socioeconomic status in Iran was made in 2008 for Tehran City. Although it was useful, due to limitations of scope as well as restrictions in its usage in different societies with different cultural, environmental, demographic structure and their changes overtime it has not gained much interest.^4^ Therefore, development of an instrument that enables measuring the socioeconomic status as a standard as well as an auxiliary questionnaires for researchers in field of health research, with applicability in a wide range of societies seemed quite critical in Iran.

A large research project is being conducted in order to develop and assess validity and reliability of a questionnaire to measure Iranian households ‘socioeconomic status with an emphasis on health studies. Applicability and construct validity of the questionnaire is being assessed over a large population survey of 6000 households in five representative districts of East Azerbaijan and 1000 households from Tehran as well as250 households from 40 other districts of Iran. At the first phase of this project the questionnaire was developed and assessed in Tabriz Metropolitan, results of which is reported in present article.

## Materials and Methods

### Study design and sampling

Development and psychometric evaluation of the socioeconomic status questionnaire involved four phases assessing the reliability, structure validity, diagnostic validity, generalizability and applicability to rural populations(out of scope population)(Table1).

The present study reports phase1 conducted in Tabriz during the year 2014. At the three later phases of the study, the applicability of the questionnaire is being investigated through a large sample enrolling participants from national variety results to be published subsequently. A mixed method comprising qualitative and quantitative data collection and analysis procedures was used to conduct a cross-sectional study in order to develop, improve and finalize a multi-domain scale for assessment of socioeconomic status in the present study (phase1). It was comprised of several stages as; developing the initial version, qualitative study and feasibility evaluation; and assessment of the validity and reliability. The initial version was developed based on international experiences retrieved from literature review and modified by expert panel examination. It was then piloted in a small sample later being applied at a metropolitan level to assess the reliability and factor structure of the scale.

**Table 1:** The four phases of the research project on development and assessment of the socio-economic status scale for health research in Iran

**Phase**	**Description**	**Status**
Phase1	Development of the questionnaire and assessment of its reliability and validity at Tabriz metropolis level	Present manuscript
Phase2	Assessment of diagnostic validity of the scale to detect marginal urban populations: 1000 households	At submission
Phase3	Generalizability of the questionnaire through its application at: 1- Other cities of province: 5000 households 2- Tehran as the capital of Iran:1000 households3-Some of other cities of Iran(40 districts from all provinces: 250 households)	Data collected. At analysis phase
Phase4	Assessment of the questionnaire in rural populations	Not conducted yet

**Fig.1:**
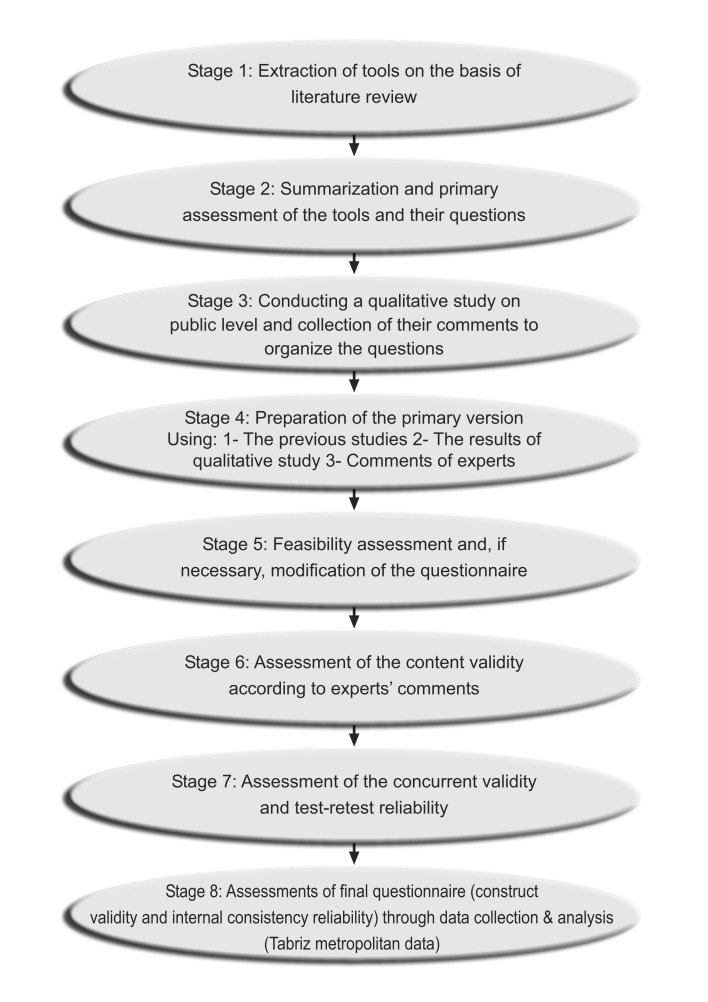
The process of development and evaluation of the socioeconomic status assessment questionnaire for Iran (SESIran)

Household was unit of sampling. The source population and target population of the study were East Azerbaijan Province and Iranian urban households, respectively. The details of development and validation of the scale is provided in Figure 1.

Cluster, purposive and simple random sampling methods were used at various stages (Figure1). Probability Proportional to Size (PPS) cluster sampling method with a cluster size of 35 households was used to enroll informant respondents from a household level sampling unit. The sample framework for selecting the clusters was based on recent governmental census.

### Development of the questionnaire

Figure1 shows the development process of the primary questionnaire at various stages. The currently available questionnaire developed in previous studies with a focus on Eastern Mediterranean Regional Office countries were identified, summarized and distributed to an expert panel including experts in field of methodology, health economics, sociology, social sciences, health administration, statistics and epidemiology & public health. The expert panel discussed over the applicability of the items from literature review. A primary scale was developed while selecting some items form the retrieved questionnaires, modifying some of them and adding new items (stages 1 & 2).Then it was assessed using by qualitative pilot in which, the purposive sampling method was used and interviews were done with different population groups individually examining the relevance of the questions to the aim of this study to refine the questions (stage 3).

Along with notes, the recorded documents were transcribed and analyzed using content analysis method performed by two persons independently. After the comparison, any ambiguities were removed with reference to texts or even participants. The interviews were continued until saturation happened and new relevant information was not collected. At stage4, the primary questionnaire was approved by experts.

Feasibility, the lack of response and other potential problems were evaluated through distribution of the questions among 40 households in Tabriz. Simple random sampling method was applied and finally the necessary reform was performed (stage 5).

### Assessment of content validity

Content validity of the questionnaire was assessed using external experts’ comments (stage 6). Ten experts in fields of methodology, health economic, sociology, social sciences, statistics and epidemiology and public health have been asked for their comments. Head of the household’s occupation was ranked according to the prestige attached by the society. Above that, we used the study conducted by Alizadeh and Rezayi^8^ and experts comments in this study (researchers) considering occupation prestige. The content validity index (CVI) was used. Scores higher than 0.75, were approved by researchers.

### Assessment of concurrent validity and test-retest reliability

The questionnaire previously developed in Tehran was used to assess the concurrent validity of the current questionnaire (stage 7). The shortened version of questionnaire includes 4 factors (head of household’s education, households spouse’s education, house and facilities, leisure time). The minimum score is 16 and the maximum is 48. Its psychometric properties have been reported earlier.^4^ The two questionnaires (Tehran questionnaire and the questionnaire designed in present study) were distributed concurrently among 30 households selected using simple random sampling method from a previous survey framework in Tabriz. The Pearson correlation test was used to assess the co-variation of the scales.

Test-retest method was used to assess the stability of the questionnaire assessed using intraclass correlation coefficient (ICC) (stage 7).

### Assessment of construct validity

Exploratory factor analysis was used to assess the construct validity using principal components and the rotation of direct oblimin methods (stage 8). Ten persons were needed for each item in the questionnaire through the exploratory and confirmatory factor analysis.^9,10^The questionnaires were distributed among 700 households in Tabriz (cluster sampling method).

Inclusion criteria were family members to live in the same residence, having same goals and features of all household members and shared family expense. Participants’ consent to be included in the study was obtained and there were no risks in responding to the questions. Exclusion criteria was separation of children from family as separate households, lack of common expenses and goals, lack of consent to participate in the study and any disorder that causes disability to answering the questions.

Confirmatory factor analysis was used in 60% of the data for exploratory factor analysis (420 households) using maximum likelihood method at stage8. The sample sizes for both exploratory and confirmatory factor analysis were more than the minimum recommended. Since there are various indicators for the assessment of the model and only one particular aspect of fitness was considered, we used some indicators. Indicators of Goodness of Fit Index, relative chi-square, Comparative Fit Index, Incremental Fit Index and Root Mean Square Error of Approximation was used. Values below 5 for relative chi-square, below 0.08 For RMSEA and above 0.8 For GFI, CFI and IFI were approved by the researchers in order to model fitting.

### Assessment of internal consistency reliability

In order to determine the internal consistency reliability of questions Cronbach's alpha coefficient was used for each factor as well as total factor separately (stage 8). A value above 0.7 for Cronbach's alpha and higher than 0.5 for ICC, were considered as acceptable reliability. The value of .05 was considered as statistical significance level for all tests.

SPSS, version 21, (Chicago, IL, USA) and Amos (version 13) software packages were used to analyze data.

The mean of normalized scores were used for scoring the socio-economic status. In order to normalize the scores a value from 0-100 was allocated to each question according to the formula (question score=x-min/max-min×100)) (x=preferred switch, min= first switch number, max= latter switch number). Average scores for each question makes up the total score and the socioeconomic status is grouped into very low, low, medium and high according to quartiles using values of total score. The questionnaire will be evaluated and upgraded every two years and the scoring method of the present questionnaire is primary and it may be changed in the next study phases (next versions) or in annual evaluation.

### Ethical consideration

When necessary, consultation and explanations about the study procedure was provided for the participants, especially for the illiterate. Then, informed consent was taken from them.

## Results

### Content validity

After distribution of the primary questions and collecting the comments from experts, necessary reforms were made using experts through simplification or replacement and means navigation in words. The results of the reforms resulted in the deletion of 3 out of 27 in total variables about health and occupation status. After redistributing among experts in field of social science, economic and health, the CVIs were reached as 0.86, 0.83, 0.82, 0.92, 0.85 and 0.90 for the total scale, main factor, self-evaluation of the expenditure capacity, wealth, house and furniture, and health expenditure, respectively.

Multiple imputation method was used in order to resolve the missing data. Data analysis was done for 30 households to assess the concurrent validity and test-retest reliability. It was also performed on the data for 700 households to assess the construct validity and internal consistency of the scale.

### Concurrent validity

The Pearson’s correlation coefficient positive (r=0.54) and significance level (*P*=0.02) between total score of the questionnaire in Tehran and the questionnaire used in this study shows that these two questionnaires are designed for the same purpose. In other words, they are targeting the same construct.

### Internal consistency reliability

Table 2 shows the internal consistency reliability. Final questionnaire includes 5 factors with Cronbach's alpha equal to 0.84, 0.96, 0.70, 0.66, and 0.55 for main factor, self-evaluation of the expenditure capacity, wealth, house and furniture, and health expenditure, respectively. Although, the Cronbach's alpha for house and furniture and health expenditure was less than acceptable value, as they have few numbers of questions and due to high value for total questionnaire, they could not be deleted.

**Table 2:** Situation of the internal consistency and test-retest reliability for each domain

**domain**	**Number of questions**	**Cronbach's alpha**	**Mean of inter-item correlation**	**Internal** **consistency reliability**	**ICC** ^*^ ** (95%confidence interval)**	**Test-retest reliability**
Main	5	0.84	0.55	Suitable	0.93(0.82 -0.97)	Excellent
Self-evaluation of the expenditure capacity	6	0.96	0.79	Suitable	0.78(0.53 -0.90)	Suitable
Wealth	5	0.70	0.41	Suitable	0.57( 0.04 -0.81)	Medium
House and furniture	4	0.66	0.42	Medium	0.68(0.29 -0.85)	Suitable
Health expenditure	2	0.55	0.43	Medium	0.83(0.63 -0.92)	Medium

Note: Total Cronbach's alpha and total Intraclass correlation were 0.91 and0.66, respectively /*Intraclass correlation

### Test-retest reliability

Table 2 shows the test-retest reliability situation for each factor separately. ICC is higher than 0.5 for all factors which is acceptable.

### Construct validity

Exploratory factor analysis was used according to different factors frequently. Ultimately, the 5-factor model was the best result of a simple pattern of loadings based on Scree test and two variables were removed in the remained analyses. A total of 69.8 percent of variance was explained by the 5-factors model (22 variables).The structure of extracted factors from correlation between the factor loadings and questions using the principal components method and oblimin rotation is shown in Table 3.

**Fig. 2:**
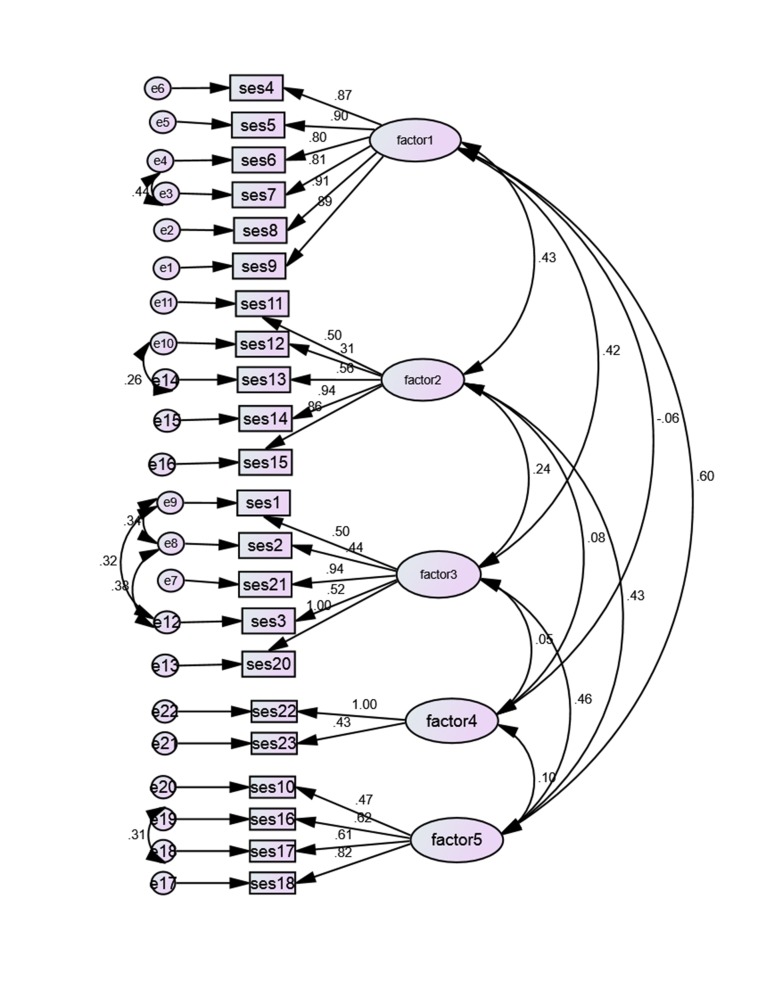
Confirmatory factor analysis

The value of Bartlett’s test of sphericity and KMO was obtained 354/9398 and 0.914, respectively. Bartlett’s test of sphericity was significant (*P*<.001). The factors i.e. Main factor, self-evaluation of the expenditure capacity, wealth, house and furniture, and health expenditure, include 5, 6, 5, 4 and 2 items, respectively. Figure 2 shows diagram of the 5-factor model.

**Table 3:** Extracted factors structure using by the principal components method and oblimin rotation

**Items**	**Factors**				
	1	2	3	4	5
Financial ability of securing the education	-0.934				
Financial ability of securing the clothing	-0.933				
Financial ability of securing the health expenditure	-0.912				
Financial ability of securing the fruits and feed	-0.905				
Financial ability of securing the ornaments and decorative accessories	-0.893				
Financial ability of securing the costs of traveling and recreation	-0.891				
Value of cash assets		0.879			
Monetary value of other assets		0.862			
Monetary value of other estate		0.722			
Monetary value of gold, jewelry, decorative accessories		0.617			
Monetary value of personal car		0.373			
Education of head of household			0.940		
Number of successful years of education			0.926		
Occupation rank			-0.754		
Income			0.644		
Occupation rank of head of household from own view			-0.618		
Share of health expenditure from total household costs				0.840	
Total health expenditure of household				0.839	
Monetary value of the carpet					0.774
Monetary value of refrigerators and TV					0.754
Monetary value of the sofa					0.751
Monetary value of housing					0.635
**Percentage of explained variance**	**40.4**	**9.2**	**8.2**	**6.4**	**5.6**

Note: The names of factors are self-evaluation of the expenditure capacity (factor 1), wealth (factor 2), main factor (factor 3), health expenditure (factor 4), house and furniture (factor 5).

Arrows drawn from the error (e) toward items show the effect of errors on the items and those, which drown from factors toward items, show that the factors are reason of items.

Factor 1 assesses economic expenditure from respondents’ view that includes items 4, 5, 6, 7, 8 and 9. Factor 2 is about households’ asset that includes the items 11, 12, 13, 14 and 15. Factor3 is named main factor includes education (2 items), occupation (2 items) and income (1 item). Factor 4 assesses the health expenditure of household that includes 2 items and factor 5 assesses the house and furniture that includes 4 items.

The factors have medial correlation. Furthermore, there is correlation between the errors of the question including 1, 2, and 3 with each other, 6 with 7, 12 with 13 and 16 with 17. Standardized coefficients of confirmatory factor analysis for this structure are presented in Figure 2. All the coefficients were significant (*P*<.05) that are shown in Table 4.

**Table 4:** Confirmatory factor analysis coefficients

			Loading	S.E.	Standardized Estimate	T Value	P-Value
SES9	<---	Factor1	1.000		0.887		
SES8	<---	Factor1	1.041	0.036	0.913	28.634	<0.001
SES7	<---	Factor1	0.982	0.044	0.812	22.341	<0.001
SES6	<---	Factor1	0.966	0.044	0.804	21.917	<0.001
SES5	<---	Factor1	1.043	0.038	0.896	27.390	<0.001
SES4	<---	Factor1	0.953	0.037	0.873	25.895	<0.001
SES21	<---	Factor3	1.000		0.943		
SES2	<---	Factor3	0.394	0.040	0.440	9.909	<0.001
SES1	<---	Factor3	0.453	0.039	0.501	11.622	<0.001
SES12	<---	Factor2	1.000		0.309		
SES11	<---	Factor2	0.930	0.167	0.500	5.562	<0.001
SES3	<---	Factor3	0.343	0.028	0.521	12.226	<0.001
SES20	<---	Factor3	0.936	0.016	0.999	57.473	<0.001
SES13	<---	Factor2	1.148	0.178	0.559	6.435	<0.001
SES14	<---	Factor2	1.440	0.230	0.939	6.271	<0.001
SES15	<---	Factor2	1.194	0.191	0.859	6.256	<0.001
SES18	<---	Factor5	1.000		0.823		
SES17	<---	Factor5	0.578	0.054	0.615	10.707	<0.001
SES16	<---	Factor5	0.618	0.057	0.624	10.860	<0.001
SES10	<---	Factor5	0.850	0.098	0.474	8.648	<0.001
SES23	<---	Factor4	1.000		0.434		
SES22	<---	Factor4	1.109	0.113	0.998	9.852	<0.001

S.E.: Standard Error

The standardized estimates indicate the items related to corresponding factor; factor 1 (self-evaluation of the expenditure capacity), factor 2 (wealth), factor 3 (main factor), factor 4 (health expenditure), factor 5 (house and furniture). Standard error has been reported and the statistical significance was established through an examination of the t value.

The results of confirmatory factor analysis showed that the 5-factor model has a good fit with the data (Chi2/df=3.9<5). Other indices, also, confirmed this fitness (CFI=0.90, GFI=0.87‏، IFI=0.91 and RMSEA=.08, 90%CI= (0.07, 0.09)

## Discussion

This study presents a questionnaire that includes 5 factors: main factor, self-evaluation of the expenditure capacity, wealth, house and furniture, and health expenditure. According to the statistics of validity and reliability observed in this study, the questionnaire presented and named as SESIran (version U1) is a valid and reliable questionnaire for assessing SES in similar urban settings.

### Main factor

It has been proven that assessment of socioeconomic status is difficult in flexible and diverse society but it has been found that occupation is the best single predictor for its determination and occupational status has high stability.^11^ For this reason, the occupation was used as one of the impressive factors on socioeconomic status based on occupational rank using by occupations prestige. Determination of the prestige assigned to occupations is a key problem. In this study, we tried to resolve some of limitations of the previous studies (set of occupations on one level that are not at the same level concerning occupation power, lack of separation of occupations accurately). For example, Alizadeh and Rezayi^8^ placed all the people who had military occupation in a class while this study took in to consideration military servicemen’s rank in classing them. In other words, people with different position or military rank are assigned to different divisions. Furthermore, the jobs that were not considered in previous studies and researchers came across to them during the data collection in this study, were ranked. As a result, objectively and referring to the previous related studies especially to the study done by Kazemipur^12^ occupational prestige was defined. In addition, income and education are known as impressive factors on socioeconomic status and the three variables (occupation, education and income) are used in this study frequently.^5,6,13^While these factors by themselves or altogether were used as the socioeconomic status determiner in some studies.^14,15^ Therefore, the criticism that can be leveled is that they did not consider all three factors while these were loaded in a main factor in the present study.

The education has been used as ordinal variable (level of education) in most of the indicators used inIran.^4,16^ In addition, survey of the evolution of the new system of education in Iran shows that social adaption of innovation is not considered in reconstruction and reform of the system. In addition, the innovation have been imposed on the educational system by directive, resulting in the success rate of innovation on entering the classroom and teaching-learning process to be low in many situation in Iran.^17^ The pre-academic educational structure and classifications has been reformed several times through the past decades, leading the classification and ranking of the levels of non-academic education in Iran to be quite inconsistent. Therefore, to overcome this limitation, one item that was loaded into the main factor was considered successful years of education. This was used in developed countries as well.^5,18^

On the other hand, according to the latest statistics of higher education in the country in 2013, the number of associate and bachelor students were much larger than higher degrees^19^ and the chances of obtaining the first four years of academic education is different from higher degrees in terms of difficulty. For this reason, in rank order classification of the number of successful educational year, these courses were considered as one category to measure socioeconomic status more accurately.

### Self-evaluation of the expenditure capacity

In developing countries due to the possibility that people are unwilling to respond to questions about their income and weak registration system on income and taxing measuring income is difficult and less reliable than high-income countries. Effort has been made to resolve the problem focusing on expenditure factor to resolve the problem. For example, a questionnaire that was updated in Egypt, in 2012, did not use questions, which directly enquired about their income in monetary unit, instead used items in which, the term money was absent.^7^ In pure economics studies, questionnaires can be found which can study expenditure factor thoroughly. Although using these questionnaires in pure economic studies can be completely justified, using them especially in large-scale health studies is impractical and not cost-effective. Moreover, through assessment of expenditure will lead to development and use of long checklists or questionnaires. As a solution, specific strategy used in this study, lacking in previous studies was that we measured expenditure capacity instead of expenditure details. This has been started and improved by the authors in few previous studies prior to using it as a section in SES assessment questionnaire.^20,21^ There was acceptable internal consistency in assessing the expenditure capacity being high in this study (0.96) and acceptable in the afore mentioned studies.

### Wealth, house and furniture

Although the studies that have used the above mentioned factors, they may not fully study the potential and important effects of socioeconomic status on health.^18^ There is extensive strong evidence that explains the effects of wealth as an important factor on socioeconomic status and it cannot be index of income. For example, it can be buffered for income in social groups that have no income (due to diseases or unemployment) or have equal income and it can be used in the form of 1-item or multi-items.^18^ We used wealth-using questions about households’ wealth or house and furniture extracted in two separate factors and they are similar to the questionnaire constructed in other countries.^4,7,22^

Details of wealth type such as refrigerator or TV brand had been used in previous studies but because the varieties of household appliances are expanding and their price gaps are reducing, it may lead to inefficient assessments. For this reason, the researchers used wealth based on prices of appliance. An advantage of this is the ability to update the wealth, if, over time; economic changes affect the value of the assets according to the economic indices.

### Health expenditure

We used health expenditure that it is not converted to wealth. For example, the one who spends his or her expenditure for their health is not considered their wealth. This is a strong point of this questionnaire.

At first, 28 variables were included for the questionnaire of interest but due to some ambiguous questions, which came into the light during the interview with people groups, 4 of them were deleted. Its content validity was confirmed both qualitatively and quantitatively. Other index that was used in order to assess concurrent validity was Pearson correlation coefficient. Moderate correlation between the scores of the two questionnaires, which confirms utility of the validity, was reached. Due to lack of a standard questionnaire, this type of validity has been overlooked in previous studies. Intra-class correlation coefficient showed that the questionnaire has high stability. Additionally, Cronbach's alpha confirmed internal consistency for total questionnaire and sub-scales. Ultimately, confirmatory factor analysis approved the fitness of the model to data.

One of characteristics of this questionnaire is that its questions are easy and transparent. In addition, we used encoded jobs in order to obtain more accurate response to the questions on job rank. Another characteristic of SESIran is that unlike the questionnaires that are specific to economy, it is short and does not require professional interviewers.

Although this questionnaire includes 22 items and can be used in research as a principal or secondary questionnaire, but in some studies, which have limitations in data collection and implementation a shorter version of the questionnaire, is needed. Accordingly, in phase 2 of the source project the researchers made an effort to provide a more shortened version of the questionnaire to study the socioeconomic factor. The primary version of the questionnaire, which covers Tabriz population alone, is presented in the current article. In next phases of the study, larger populations will be studied and new versions of the questionnaire will be provided for interested readers that can be used for wider populations.

### Limitations

Due to time and cost restrictions, the researchers did not apply this questionnaire to rural setting or make modifications to provide a rural version. This provides an agenda for future studies to evaluate this questionnaire in rural areas of Iran and, if necessary, adapt a rural version. Not exceptional to this study, a general limitation of assessing economic status in many developing countries is that some people may think that declaring higher income may affect their future financial benefits. To reduce this effect, the questionnaire was designed on a basis to diminish the role of direct items assessing income level. The interviewers were also trained to inform clearly and honestly about the objectives of the study and confidentiality of the collected information. To ensure the generalizability of the developed questionnaire, a multi-phase study was designed and the current phase provides the preliminary version that may be improved over the subsequent phases of the source project.

## Conclusion

The questionnaire developed in present study appeared to be a valid and reliable SES assessment tool. It could be of value to be used not only as a complementary questionnaire in most health surveys or clinical studies, but also as a main questionnaire in health equity and health economics research.

## Acknowledgment

This article was conducted as under a thesis grant for degree of master in Epidemiology at Department of Statistics & Epidemiology, Tabriz University of Medical Sciences.

## Competing interest

The authors claim no conflict of interests with other people or organizations
